# Extension for Prevention

**Published:** 1901-06-15

**Authors:** Wm. H. Trueman

**Affiliations:** Philadelphia, Pa.


### EXTENSION FOR PREVENTION.

BY DR. WM. H. TRUEMAN, PHILADELPHIA, PA.

The advocates of extension for prevention have accepted the latest suggestions of laboratory experiments regarding the initial cause of dental caries, and contend that maintained cleanliness of the cavity margin must be added to other accepted requirements. They therefore insist that the marginal lines should be placed beyond doubtful territory; they insist that cavities should be so extended that the filling presents throughout this entire territory a smooth, highly polished surface of gold, replacing with gold the more vulnerable tooth tissue, and advise extensive sacrifice of sound tooth tissue if needful to attain this end.

This position, if the premises upon which it is based are true, is unassailable. Whether the extension for prevention is much or little is beside the question. If the future safety of carious teeth demands that the gingival margin in approximal cavities should be extended so as to lie under the gingival gum septa, and should also be further extended sidewise until a position is reached unfavorable to the undisturbed lodgment of deleterious matter, anything short of this and more than the removal of carious and uncertain tissue and such sound tissue as may be necessary to make the cavity accessible and retentive in shape, is a useless waste. Extension for prevention, if necessary, should be thoroughly done. If it is necessary, the needful skill and all that is required on the part of the operator the past has abundantly proved our profession can supply. If it is the best, those who want the best, and are willing to pay for the best in patience, endurance and cash, will find in our profession that want fully met. We leave out of the count the typical "nervous" patient, those unfortunates so gingerly put together that they should never have been born, or having been born, should have died before their first teething period. I approach the question, considering those cases only where it is, in its fullest sense, practicable, and narrow it to the one thought, "Is it necessary?" Is it the best thing to do for the salvation of the affected tooth?

The fact that teeth in the same mouth are more liable to decay when not filled than when filled, although the filling may be a small one, I hold has no bearing on this matter. All the teeth in same mouth are liable to the same incidents and accidents, especially corresponding teeth of the same jaw. They have, very nearly the same surroundings, and are, therefore, subject to like defects and injuries. While these predisposing and exciting causes are common to all the teeth in the same mouth, they vary potentially. Some teeth predisposed break down early, some later, and some not at all. If the small filling remedies the defect, the decay is arrested; if it fails to do so, decay will recur.

It will require a very much longer time than has yet elapsed since the idea of extension for prevention was first promulgated to determine whether immunity from recurring decay bears a marked relation to the area embraced by the filling or not. We may remember, and perhaps with profit, the advent and the exit of separation for prevention nearly a generation ago. You know, however, how quickly it proved disastrous when the profession at large put it to a crucial test. In the hands of a few, who mastered its technics and learned its limitations, it still continues a tooth saving practice.

In common with extension for prevention, it called for the destruction of sound tooth structure; it differed in that it aimed to make the vulnerable surface self-cleansing, while extension for prevention ignores the presence of deleterious matter and seeks to render it inert for harm by replacing the natural and destructible surface against which it rests with one artificial and resistant. If the practice proves as excellent as its advocates promise, may we not soon be called upon to prevent by anticipation?

It is the dentist's mission to save the teeth and prolong their usefulness to the utmost in all the varied capacities they serve. It is not derogatory to initiate or practice methods that simplify, or make it easier or more comfortable for patient and operator. We elevate the profession as we increase its usefulness to the people whom we serve. To insist that all operations should be, artistically and mechanically, "as perfect as the hand of man can make them," regardless of the fact that but few can so serve or be served, may elevate the profession as a body of artists and artisans, but curtails greatly its usefulness as a health and comfort promoting fraternity.

The simple fact that comparatively few of the vast number of reparative dental operations reach that high standard while so many prove effective tooth savers gives point to the question, "Is it necessary?"—*Items of Interest*.

*Formaline*.—Formaline has properties which give it value in certain cases. It is a powerful germicide, and tans albuminous or gelatinous matter. It is somewhat volatile at ordinary temperatures and will diffuse itself when applied to soft tissue. Thus it can be used to sterilize the periapical region to some extent if used in proper dilution and moderately applied. It can not be used in full commercial strength with safety on soft tissues, but sparingly in pulpless root canals. It is terribly irritating and destructive to living tissue, not as a cauterant but as a chemical poison. It may be used in from a 1 to a 2 percent solution in dental disinfection. A 5 or 10 percent solution is valuable as a hardening fluid for histological or pathological tissues, especially when combined with bichlorid of mercury.—Dr. E. C. Kirk, in *International Dental Journal*.

